# Moisture Content Distribution in Cross-Section of Cylindrical Wood Components

**DOI:** 10.3390/polym17222994

**Published:** 2025-11-11

**Authors:** Panpan Tian, Heng Zhang, Jianhong Han, Yu Zhao, Xia Han

**Affiliations:** 1 School of Civil Engineering and Architecture, Xinjiang University, Urumqi 830047, China; 2 School of Civil Engineering, Xinjiang Institute of Engineering, Urumqi 830023, China; 3 China Energy Engineering Group, Hunan Electric Power Design Institute Co., Ltd., Changsha 410007, China

**Keywords:** wood components, moisture content distribution, average moisture content, moisture content gradient

## Abstract

The moisture content of wood components varies with changes in the external environment, which significantly affects the mechanical properties, moisture stress, decay, drying shrinkage, and cracking of wood components. Therefore, calculating the moisture content distribution of the cross-section of wood components is an important basis for in-depth research on wood components. First, a hygroscopicity test was performed on 45° sector-shaped Chinese fir thin-plate specimens. The specimens were treated to an absolutely dry state and placed in two different environments. The average moisture content and moisture content gradient on the cross-section of the specimens were measured, and the spatial distribution and temporal variation in the moisture content were studied. A theoretical model for the moisture content distribution of wood was then derived based on food drying theory. Finally, the applicability of the theoretical model was verified through experiments, and the effects of the root order *μ*_n_ of the characteristic equation of key parameters, the size of the component, and the position of the component on the moisture content distribution were discussed for the theoretical model. During the hygroscopic process, the average moisture content of wood components increased continuously, but the growth rate gradually slowed. The surface moisture content rapidly reached the level of the external moisture content first, followed by the equilibrium moisture content within a few hours. Hygroscopic hysteresis evidently occurred within the wood, which may take dozens or even hundreds of days. When calculating the average moisture content model of cylindrical components, as well as those of the models of the spatial and temporal variation in the moisture content, it is sufficient to take the first 3 orders of the root *μ*_n_ of the characteristic equation of the first Bessel function *J*. The rate of moisture release of cylindrical components is faster than that of laminates because the ratio of the surface area to the volume of a cylinder is greater than that of a plate, and the former is twice that of the latter. The results revealed that the theoretical model for the moisture content distribution of wood has good accuracy and applicability.

## 1. Introduction

Moisture content is one of the most critical timber parameters [1]. Timber is hygroscopic, and its moisture content dynamically responds to variations in ambient environmental factors, including relative humidity, temperature, and precipitation. The spatial distribution pattern of moisture content within timber is of particular significance. This distribution characteristic of timber is fundamental for conducting in-depth analyses of the basic physical and mechanical properties [2], moisture-induced stresses [3], shrinkage cracks [4], creep properties [5], and decay processes [6], among others. These insights are essential for optimizing timber processing techniques, enhancing structural design, and ensuring the durability of timber-based products.

Numerous scholars have conducted research on methods for testing the wood moisture content. Wang [7] designed an intelligent wood moisture content tester using the resistance method based on the material properties of wood, which improved the automation level and testing accuracy of wood moisture content detection. Building on this foundation, Fredriksson et al. [8] developed a small resistive wood moisture content meter and experimentally determined its precise measurement range. To further enhance the accuracy of measuring the wood moisture content using the resistance method, Hasfa et al. [9] measured the moisture content of the cross-section of pine wood using both the resistance and finite element simulation methods. They then obtained the relationship between the resistance and moisture content of wood under constant-temperature conditions. In addition to the embedded resistance method, Uwizeyimana et al. [10] investigated the use of surface-type electrodes for measuring the moisture content of glued laminated wood. They established a calibration curve, which improved the accuracy of the surface-type electrodes in measuring the wood moisture content. Brischke et al. [11] developed a robust, precise method for measuring wood moisture content using materials such as electrodes, conductive adhesives, and insulating adhesives. Liu [12] proposed a method for detecting wood moisture content using stress waves based on the specific gravity of wood fibers. He established a theoretical model for the longitudinal propagation velocity of wood and verified its accuracy experimentally. Mai et al. [13] proposed a nondestructive method for predicting wood moisture content using ground penetrating radar frequency, based on the correlation between the dielectric constant of wood and its moisture content. Machine learning has emerged as a powerful predictive framework. Rahimi et al. [14] used gradient-boosting models to estimate internal moisture content gradients in kiln-dried timber, leveraging wood attributes and drying parameters. Time-domain nuclear magnetic resonance (TD-NMR) (Suekuni et al.) non-invasively tracks microscale water distribution during drying, capturing fluid–solid interactions unseen in bulk measurements [15]. The reconstructed images from micro-computed tomography (micro-CT) scans have been verified by Scholar Silva et al. as an effective tool for this type of evaluation [16]. Near-infrared hyperspectral imaging (Awais et al.) maps surface moisture content distribution, even distinguishing earlywood-latewood differences in acetylated wood [17]. Furthermore, new technologies, such as computed tomography [18], laser scanners [19], and nuclear magnetic resonance [20] have been applied to research on inferring wood moisture content, providing new technical approaches and research directions for the development of this field. These techniques form a multi-scale framework to advance wood moisture content detection, optimizing processing and product performance.

Several scholars have also investigated the distribution of wood moisture content. Chiniforush et al. [21] measured the moisture content of wood using the weighing method, obtained the adsorption isotherms of wood, fitted the analytical equations, and determined the relationship between the diffusion coefficient of wood and temperature as well as moisture content. Chen et al. [22] conducted experimental and finite element studies on the moisture content distribution of Anhui fir. The fitting results demonstrated that the moisture content of wood exhibits a quadratic distribution on the cross-section and an exponential distribution over time. Svensson et al. [23] aimed to predict the changes in the moisture content of wood components under continuously changing climatic conditions. They leveraged the factors affecting the moisture content of wood components in a fully coupled transport model, established a theoretical model for the changes in the moisture content of wood under continuously changing climates based on Fick’s second law, and verified and supplemented the theoretical model through the finite element method. Niklewski et al. [24] established a numerical model for predicting the moisture content of wood after rainfall based on the surrounding environment by utilizing Fick’s second law. Zhou et al. [25] employed the finite element method to establish a mathematical model for the spatial distribution of wood moisture content and density and verified the accuracy of the model and the feasibility of using the finite element method to study the wood moisture content. Autengruber et al. [26] proposed a method for estimating the moisture content distribution of larger cross-sections based on relative humidity. Wang et al. [27] adopted a finite element method with nonlocal boundary conditions to study the nondestructive testing of wood moisture content based on a model of planar capacitance sensors and fitted the relationship between the capacitance of wood and its moisture content. He et al. [28] established a heat and mass coupling transfer model for wood during drying, which can effectively predict the changes in the moisture content of wood during vacuum drying. Autengruber et al. [29] established a coupling model in ABAQUS that can simultaneously handle the changes in the content of bound water, water vapor, and free water in wood. Using this model, the moisture transfer of free water above the fiber saturation point in wood was obtained. Afshari et al. [30] compared the simulation results of ANSYS Workbench© with experimental data to study transient moisture transfer in wood and bamboo composites and determined the influence of wood, bamboo, and adhesives on the moisture transfer of the composites. Xia et al. [31] established an identification model for a wood moisture content detection system using a deep learning method and verified the accuracy of the deep learning model. Daassi-Gnaba et al. [32] studied the relationship between the dielectric constant of wood and its moisture content. Elustondo et al. [33] reviewed wood drying advances, highlighting that understanding moisture content movement, its links to drying stress and collapse, and integrating AI for process control are key to optimizing schedules and reducing defects. Hajian et al. [34] investigated species-specific moisture content dynamics via in situ X-ray CT. They found that western hemlock dried with spruce or pine schedules showed slower capillary drying, severe moisture content non-uniformity, and failed transition and diffusion phases, which were attributed to its high green moisture content and wet pockets, highlighting the need for tailored drying protocols. Accordingly, they constructed a prediction model for wood moisture content using machine learning techniques and feature selection methods and verified the accuracy of the model using experimental data.

Despite extensive research on the moisture content of wood components being conducted by numerous scholars, several critical issues remain unresolved. First, most existing studies have focused on testing techniques and methods for measuring the moisture content of wood components while the moisture content distribution characteristics remain under explored. Second, the research on the moisture content distribution across the cross-section of wood components has predominantly relied on fitting experimental data patterns or finite element analysis, with a noticeable scarcity of in-depth theoretical investigations. Third, validation for models on the moisture content distribution of wood component cross-sections remains significantly scarce. Fourthly, current moisture content distribution models for wood components are still insufficient in effectively analyzing the average moisture content and changes in moisture content gradients through precise analytical equations. Cylindrical wood components are among the most common load-bearing elements in buildings. Therefore, theoretical research on the moisture content distribution of their cross-sections is vital. To fill these gaps, this study focuses on the theoretical derivation of a moisture content distribution model for cylindrical wood components, which is subsequently validated through experiments. The developed moisture content distribution model for wood components comprehensively considers both the temporal evolution and spatial variation in moisture content.

## 2. Experimental Study

### 2.1. Experimental Design

To analyze the internal cross-sectional humidity distribution law of cylindrical components with changes in external moisture content, this study designed eight groups of water absorption tests for fan-shaped wood specimens. The tests were performed from two aspects: the average moisture content of the components and the moisture content gradient. These experiments featured variables such as relative humidity, equilibrium moisture content, and component size in their design.

In engineering practice, moisture exchange in cylindrical components occurs on the circumferential surface, whereas the two cross-sections are isolated from moisture exchange. To prevent the cylindrical specimens from cracking due to humidity changes, fan-shaped specimens were chosen as substitutes for the original cylindrical components on the premise that the boundary conditions for moisture exchange remain consistent.

**Figure 1:**
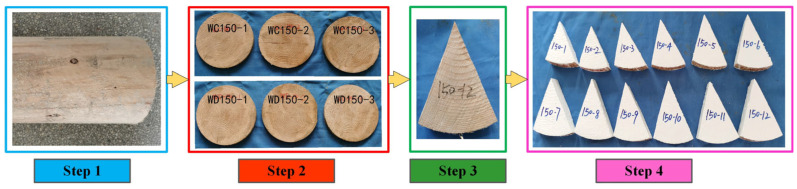
Manufacturing steps of fan-shaped specimens.

This study ensured that moisture within the fan-shaped specimens diffused radially. To prevent moisture from being absorbed by the four side surfaces of the specimens during moisture absorption, a moisture isolation material needs to be applied to the surfaces of the specimens where no humidity transfer is allowed. In this experiment, fan-shaped specimens with an included angle of 45° were employed to investigate the moisture content distribution. The cutting method of the fan-shaped specimens is illustrated in Figure 1, which can be divided into four main steps: preparing the log, slicing it into discs, cutting the discs into fan shapes, and applying an isolation layer. Chinese fir was selected for the experiment in this paper. The logs had radii of 100 mm and 150 mm. To accommodate subsequent operations such as specimen polishing, the length of the specimens was set to 40 mm.

All the specimens were dried and treated multiple times in the early stage to ensure an initial moisture content of 0%. In this experiment, two levels of the external equilibrium moisture content were set. The temperature was maintained at 30 °C with relative humidity levels of 50% and 70%. According to the equilibrium moisture content calculation formula presented as Equation (1) [22], the equilibrium moisture contents under the two environmental conditions are 8.87% and 12.78%, respectively, where *T* is the ambient temperature (°C) and *RH* is the relative humidity (%).

(1) $$w_{e}=0.01{\left[\frac{-\left(T+273.15\right)\ln\left(1-RH\right)}{\left(0.13{\left(1-\left(T+273.15\right)/647.1\right)}^{-6.46}\right)}\right]}^{\frac{{\left(T+273.15\right)}^{0.75}}{110}}$$

**Figure 2:**
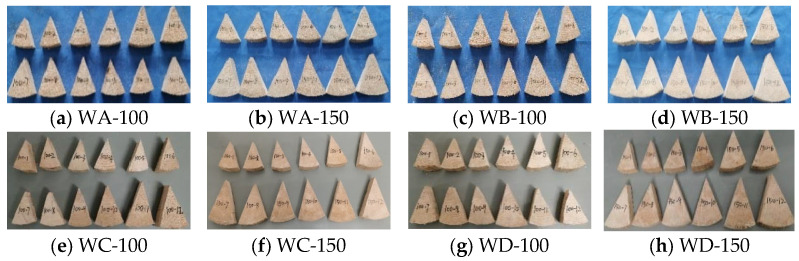
Experimental specimens.

Two specimens of different sizes were placed under two different environmental conditions for testing. The average moisture content and moisture content gradient of the wood specimens were tested under changes in the external moisture content. Eight groups of tests were performed, with each group consisting of 12 specimens, as listed in Table 1 and Figure 2.

### 2.2. Permeability Experiment

**Figure 3:**
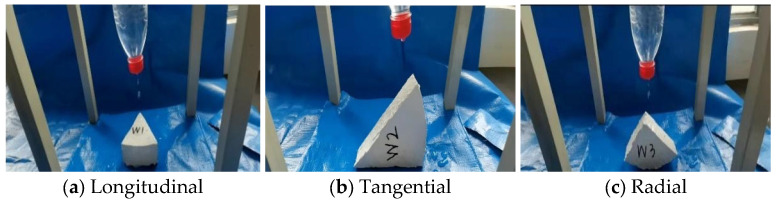
Permeability experiment.

To test the isolation effect of the isolation material, a water permeability test was performed before the measurement test. In this paper, the water permeability tests were conducted in the longitudinal, tangential, and radial directions of the test specimens, as shown in Figure 3. The isolation effect of the paint was judged by comparing the mass difference of the test specimens before and after the test. The surface pressure of the specimen is approximately 101 kPa. The test time was 30 days.

The mass results of the test specimens before and after the test are listed in Table 2. Noticeably, after the water permeability test for one month on test specimens W1, W2, and W3 with the isolation layer, the mass increased by approximately 1%, indicating that the isolation material has a good isolation effect and meets the test requirements.

### 2.3. Measurement Scheme

**Figure 4:**
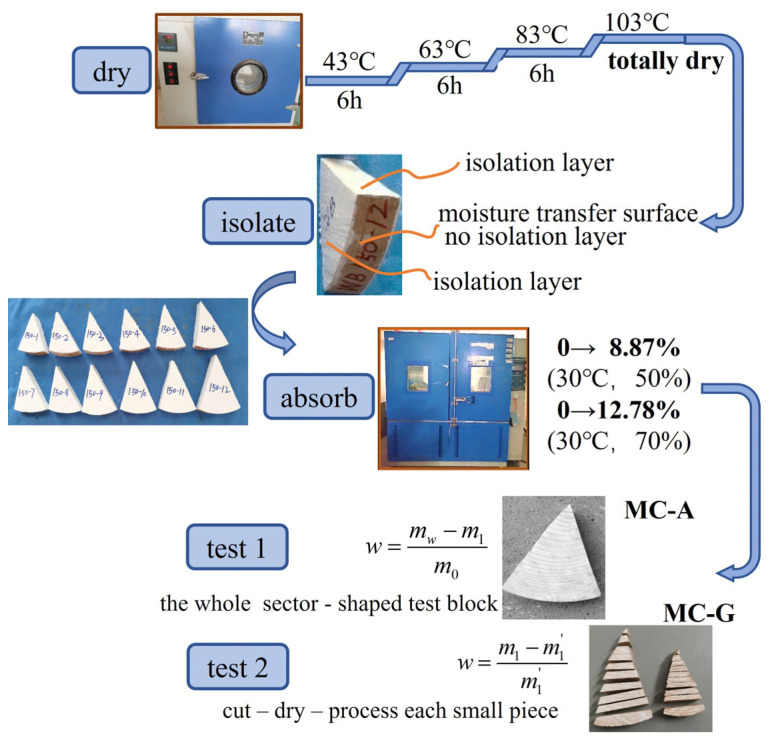
Testing of the wood specimen.

To study the distribution law of the moisture content of wood, tests were performed to determine the average moisture content of wood and the gradient distribution of the moisture content of wood. The test process mainly involves four steps: drying, isolation, moisture absorption, and testing, as shown in Figure 4.

The first step is drying. To better simulate the moisture absorption process, the initial moisture content of the entire cross-section of the wood component should be made uniform. During actual drying, in the initial stages, the sudden increase in temperature can cause some fan-shaped test specimens to experience surface cracking. Therefore, the temperature should initially be increased very gradually instead of being directly raised to 103 °C. After drying, the absolutely dry mass of the test specimen needs to be weighed. Through repeated experiments in this study, the method of gradually increasing the temperature at 43 °C (for 6 h), 63 °C (for 6 h), 83 °C (for 6 h), and 103 °C (until completely dry) is adopted. When the difference between the last two weighings of the component does not exceed 0.5% of the mass of the test specimen [34], the component is considered to have reached the completely dry state, that is, the moisture content of the cross-section is 0.

**Figure 5:**
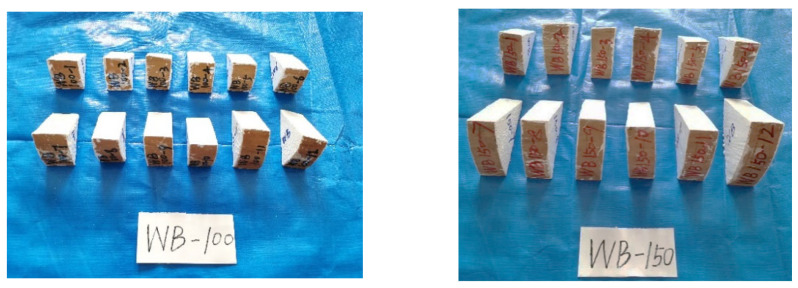
Diagram of the components after painting.

The second step is isolation. To simulate moisture exchange in log components in actual engineering projects, the circumferential surface of the log component is set as the moisture exchange surface, and the two fan-shaped cross-sections and the two side surfaces are set as the moisture isolation surfaces. After the moisture exchange surface is dried, treatment is avoided to ensure the radial humidity transfer of the cylindrical component. The moisture isolation surface needs to be adequately coated with isolation material. The isolation material used was fluorocarbon paint, and its isolation effect was introduced above. It is assumed that no direct humidity transfer occurred between the moisture isolation surface and the external environment. The painted components are shown in Figure 5.

The third step is moisture absorption. The processed test specimens are placed in a constant temperature and humidity environmental test chamber for moisture absorption. The two moisture absorption conditions are introduced in Table 1.

The fourth step is testing. Test 1 involves the average moisture content (MC-A), which comprises groups WA-100, WA-150, WC-100, and WC-150. The oven-drying method is used to measure the average moisture content [34]. Record the absolute dry mass *m*_0_ of the wood. After painting, the mass of the test specimen is *m*_1_. After placing it in the environmental chamber, measure the mass of the component at a certain measurement time point and obtain *m_w_*. The average moisture content of the component at this time point then becomes

(2) $$w=\frac{m_{w}-m_{1}}{m_{0}}$$

**Figure 6:**
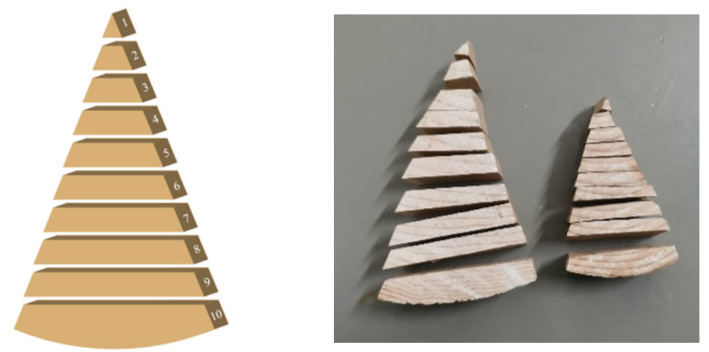
Cutting method of the specimens for MC-G test.

Test 2 involves the gradient moisture content (MC-G), which includes groups WB-100, WB-150, WB-100, and WB-150. The specimen conditions and the oven-drying test method for the moisture content gradient distribution test are the same as those for the average moisture content distribution. However, it is only necessary to measure the humidity gradient at the initial, middle, and final stages of the test. In this paper, the cutting method was used to measure the moisture content gradient of the component. After the specimen was removed from the constant temperature and humidity test chamber, a grinder was first used to grind off the paint layer on the surface of the component. Each specimen is evenly cut into 10 parts, following the cutting method shown in Figure 6. Specifically, the steps are as follows. Mark each cut specimen and weigh the mass of each small part after cutting. Then dry each small part in a constant temperature drying oven to an absolutely dry state and record the mass. Thus, the distribution curve of the moisture content gradient can be obtained. Weigh the cut pieces and denote them as *m*_1_, *m*_2_, *m*_3_, …. After weighing, dry the cut pieces in a constant temperature drying oven. Denote the absolutely dry masses as *m*_1_′, *m*_2_′, *m*_3_′, …. The moisture content of the small piece at this time point then becomes

(3) $$w=\frac{m_{1}-m_{1}^{’}}{m_{1}^{’}}$$

Among them, the moisture content gradient is only measured thrice for each group. Each time, only four specimens are selected for measurement, and the remaining specimens are kept in the constant temperature and humidity test chamber until the next cutting and then removed to repeat the above operations.

## 3. Experimental Results

### 3.1. Average Moisture Content

**Figure 7:**
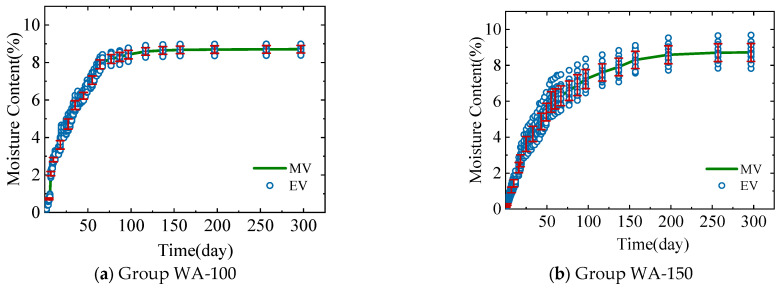

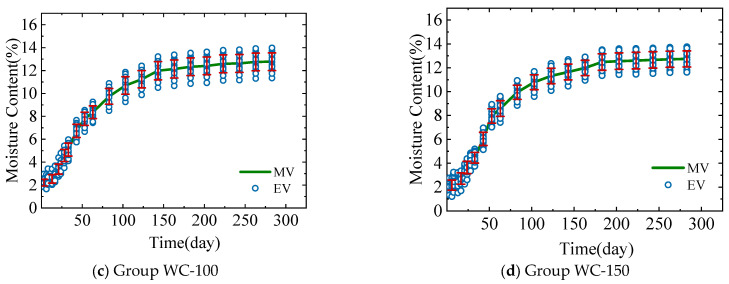
Distribution of average moisture content with time.

In the experiment, the changes in the average moisture content of wood components in Group WA-100, Group WA-150, Group WC-100, and Group WC-150 over time were measured. The four groups of experiments represent two equilibrium moisture contents (restricted by different environmental conditions) and two sizes. The changes in the moisture content values of the 12 specimens in each group over time are shown in Figure 7. EV denotes experimental value, and MV represents the average moisture content distribution curve of the 12 data points (mean value).

Figure 7 shows that, under constant external temperature and humidity, the average moisture content of the four groups of disc specimens gradually increases from 0 to the equilibrium moisture content set by the environment. In the early stage, the moisture content curve rises rapidly, indicating a fast moisture absorption rate. In the later stage, the curve gradually becomes gentle, indicating a decrease in the moisture absorption rate because the main driving force for the moisture absorption rate is the moisture content gradient. Notably, the difference between the moisture content of the wood components and the equilibrium moisture content of the external environment is greater in the early stage than in the later stage.

Comparing (a) to (b) and (c) to (d) in Figure 7 shows that, under the same environmental conditions, disc specimens with different radii take different times to reach the equilibrium moisture content. The WA-100 group takes approximately 87 days to reach the equilibrium moisture content, the WA-150 group takes approximately 137 days, the WC-100 group takes approximately 143 days, and the WC-150 group takes about 163 days. Therefore, the smaller the radius, the shorter the time required to reach the equilibrium moisture content. Moreover, in the early stage of the experiment, the curves of the moisture content changing with time for specimens with a radius of 100 mm (WA-100, WC-100) are steeper than those for specimens with a radius of 150 mm (WA-150, WC-150). Thus, the moisture absorption rate of disc specimens with a smaller radius is considered to be faster than that of specimens with a larger radius in the early stage of the experiment.

A comparison of (a) to (c) and (b) to (d) in Figure 7 shows that when the radius is the same, the time taken for each group of specimens to reach the equilibrium moisture content is different. The time taken for each group to reach the equilibrium moisture content suggests that the smaller the difference between the initial moisture content of the specimen and the equilibrium moisture content, the shorter the time taken for the specimen to reach the equilibrium moisture content. Moreover, for specimens with larger differences in moisture content, the average moisture content distribution curve is steeper in the initial stage; that is, the growth rate of the moisture content is higher.

### 3.2. Moisture Distribution in Space

**Figure 8:**
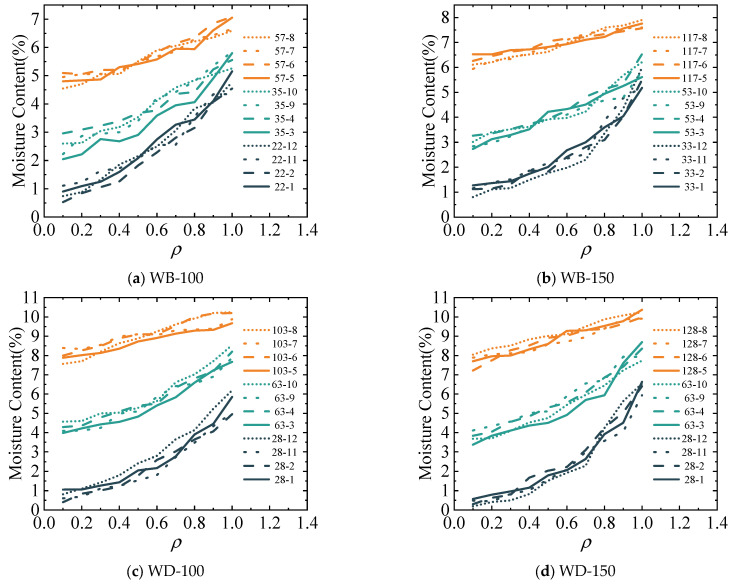
Gradient of moisture content with time.

The spatial distribution curves of the moisture content of the specimens in the WA-100 group, WA-150 group, WC-100 group, and WC-150 group measured in the experiment are shown in Figure 8. The focus during the experimental test was on the early, middle, and final stages. When measuring the moisture content gradient of the four groups of specimens, four specimens numbered 1, 2, 11, and 12 in each group were selected in the early stage, four specimens numbered 3, 4, 9, and 10 in each group were selected in the middle stage, and four specimens numbered 5, 6, 7, and 8 in each group were selected in the later stage. The number represents the test time and serial number of the specimen. For example, 35-10 in the WB-100 group represents the moisture content of specimen number 10 in the WB-100 group when the test duration is 35 days. The abscissa refers to the relative distance of the cross-section. Specifically, it is the ratio of the distance from a certain position to the pith to the distance from the outermost layer to the pith.

Figure 8 shows that, before the specimens in each group reach the equilibrium moisture content, the surface moisture content on the cross-section at any moment is greater than the internal moisture content, and the moisture content shows a decreasing trend from the surface to the interior of the component. During moisture absorption, in the early stage, it is easier for the surface of the specimen to exchange humidity with the environment than the interior. Therefore, the moisture content of the specimen surface increases rapidly, whereas that of the interior changes slowly. In the later stage, the difference between the surface moisture content of the specimen and the equilibrium moisture content decreases continuously. The difference between the internal and surface moisture contents of the specimen is greater than that between the surface moisture content of the specimen and the equilibrium moisture content of the environment. The moisture absorption rate of the specimen surface is slower than the rate at which moisture migrates from the specimen surface to the interior, and the moisture content gradient decreases to some extent.

### 3.3. Moisture Distribution over Time

**Figure 9:**
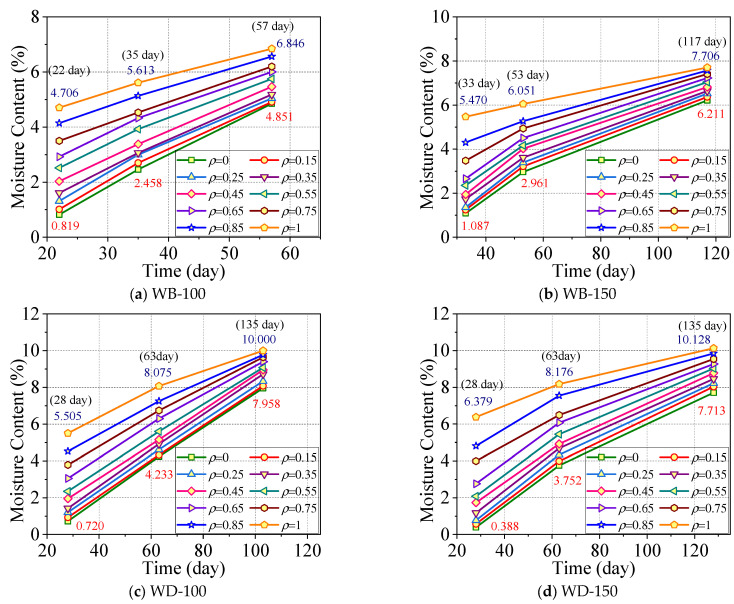
Gradient of moisture content with time.

Similarly, the moisture contents of the specimens in the WB-100 group, WB-150 group, WD-100 group, and WD-150 group were measured experimentally. The moisture content curves at the same position over time are shown in Figure 9.

Figure 9 demonstrates that the moisture content of the specimens in each group increases during moisture absorption. In the early stage of moisture absorption, the moisture content of the specimen surface increases rapidly and quickly approaches the equilibrium moisture content, but the internal moisture content is relatively low. This indicates that it takes some time for moisture to diffuse from the specimen surface to the interior. In the middle stage of moisture absorption, the moisture content of the specimen surface in the early stage has already approached the equilibrium moisture content. At this stage, the change in the surface moisture content is relatively small, and the internal moisture content gradually increases from the initial low value, but it is still less than the surface moisture content value. In the final stage, the internal moisture content still gradually increases, and the difference between the surface moisture content and the internal moisture content gradually decreases. The average moisture content of the entire cross-section is closer to the equilibrium moisture content and gradually approaches a stable state.

### 3.4. Calculation of Parameters D and S

The moisture diffusion coefficient *D* and surface emission coefficient *S* of wood are critical parameters for wood moisture transfer. According to the calculation method provided in reference [35], the values of *D* and *S* can be derived from the known moisture content change curve of wood components, and are determined using Equations (4) and (5) as follows:

(4) $$D=\left(0.049a^{2}\right)/t_{0.5}$$

(5) $$S=\frac{0.701D}{\left(a/2\right)\left[Dt/{\left(a/2\right)}^{2}-0.196\right]}$$

where *a* is the thickness of the wood, for wood with a circular cross-section, this refers to the wood’s diameter. *T*_0.5_ is the time required to reach half of the total adsorption capacity. *T* is the time required to reach the total adsorption capacity. The parameters *t*_0.5_ and *t* are determined from the curves of average moisture content versus time for each group of wood components, as shown in Figure 7. The calculation results of the parameters *D* and *S* are presented in Table 3.

## 4. Theoretical Analysis

Assuming that the moisture diffusivity *D* and the surface emission coefficient *S* are constant and the relative moisture content is substituted into the control equation of the plates, cylinders and spheres (Fick’s law of humidity transfer parameters), the internal moisture transfer of wood components follows Fick’s second law [36], expressed in Equation (6a) [37]:

(6a) $$\begin{array}{l} \frac{1}{D}\frac{\partial w(\rho ,t)}{\partial t}=\left\{\begin{array}{l} \frac{\partial^{2}\phi }{\partial \rho^{2}}=\frac{\partial^{2}(\frac{w-w_{e}}{w_{i}-w_{e}})}{\partial \rho^{2}}\\ \frac{\partial^{2}\phi }{\partial \rho^{2}}+\frac{1}{\rho }\frac{\partial \phi }{\partial \rho }=\frac{\partial^{2}(\frac{w-w_{e}}{w_{i}-w_{e}})}{\partial \rho^{2}}+\frac{1}{\rho }\frac{\partial (\frac{w-w_{e}}{w_{i}-w_{e}})}{\partial \rho }\\ \frac{\partial^{2}\phi }{\partial \rho^{2}}+\frac{2}{\rho }\frac{\partial \phi }{\partial \rho }=\frac{\partial^{2}(\frac{w-w_{e}}{w_{i}-w_{e}})}{\partial \rho^{2}}+\frac{2}{\rho }\frac{\partial (\frac{w-w_{e}}{w_{i}-w_{e}})}{\partial \rho } \end{array}\right. \end{array}$$

(6b) $$\varphi =\frac{w-w_{e}}{w_{i}-w_{e}}$$

where *ρ* = *r*/*R* is a radial variable that is the ratio of the distance from any point to the center *r* to half the radial length *R*. It is important to note that *f* is relative moisture content, expressed in Equation (6b). Symbols *w*, *w*_e_ and *w*_i_ correspond to moisture content, equilibrium moisture content, and initial moisture content respectively.

As radius 150 mm in this study is not infinite, exclude Equation (6a) for slab. Furthermore, as this study focuses on substituting thin plates for cross-sections and neglects the influence of length, it simplifies the three-dimensional cylinder into a two-dimensional model. Consequently, the spherical expression presented in Equation (6a) is also excluded.

The initial condition and boundary condition are obtained from Equations (7) and (8), respectively:

(7) $$t=0,\;w\left(\rho ,0\right)=w_{i},\;\phi \left(\rho ,0\right)=1$$

(8) $$-\frac{\partial w}{\partial \rho }{|}_{\rho =1}=\frac{S}{D}(w_{e}-w_{s})$$

In the humidity field model of wood components, the spatial distribution of moisture content, which can predict the change in the moisture content of wood components with time, is vital. According to Adebiyi’s [38] temperature field results, Equations (6)–(8) can be solved as follows:

(9) $$\varphi (\rho ,t)=\sum_{n=1}^{\infty }\frac{2Bi}{\left[\mu_{n}^{2}+Bi^{2}+2\nu Bi\right]}\left[\frac{\rho^{\nu }J_{-\nu }(\rho \mu_{n})}{J_{-\nu }(\mu_{n})}\right]\exp(-\mu_{n}^{2}Fo)$$

where *J* is the first-class Bessel Function and *μ*_n_ is the root of the characteristic equation:

(10) $$\frac{J_{0}(\mu_{n})}{J_{1}(\mu_{n})}=\mu_{n}/Bi$$

Substituting the series of the first type of the Bessel function *ν* = 0 of the cylindrical wood components into Equation (10) yields the eigenvalues, which are shown in Equation (11):

(11) $$J_{0}(x)=\sum_{m=0}^{\infty }\frac{{\left(-1\right)}^{m}}{{\left(m!\right)}^{2}}{\left(\frac{x}{2}\right)}^{2m},\;J_{1}(x)=\sum_{m=0}^{\infty }\frac{{\left(-1\right)}^{m}}{m!(m+1)!}{\left(\frac{x}{2}\right)}^{2m+1}$$

Equation (12) then describes the distribution of moisture content:

(12) $$\varphi (\rho ,t)=\sum_{n=1}^{\infty }\frac{2Bi}{\left[\mu_{n}^{2}+Bi^{2}\right]}\left[\frac{J_{0}(\rho \mu_{n})}{J_{0}(\mu_{n})}\right]\exp(-\mu_{n}^{2}Fo)$$

The center of the circle represents the neutral layer of cylindrical wood components. Substituting the boundary condition of neutral layer moisture content *ϕ* (0,*t*) into Equation (12) produces the neutral layer moisture content, which is presented in Equation (13):

(13) $$\phi =\sum_{n=1}^{\infty }\frac{2Bi}{\left[\mu_{n}^{2}+Bi^{2}]J_{0}(\mu_{n}\right)}\exp(-\mu_{n}^{2}Fo)$$

Substituting the boundary condition of surface moisture content *ϕ* (1,*t*) into Equation (12) yields the surface moisture content:

(14) $$\phi =\sum_{n=1}^{\infty }\frac{2Bi}{\left[\mu_{n}^{2}+Bi^{2}\right]}\exp(-\mu_{n}^{2}Fo)$$

The average moisture content integral of the cross-section is shown in Equations (15) and (16):

(15) $$\bar{\varphi }(t)=\frac{2}{R\theta }\int_{0}^{R}\varphi (r,t)\theta dr=\frac{2}{R}\int_{0}^{R}\varphi (r,t)dr$$

(16) $$\bar{\varphi }(t)=2\sum_{n=1}^{\infty }\frac{2Bi\exp(-\mu_{n}^{2}Fo)}{\left[\mu_{n}^{2}+Bi^{2}]J_{0}(\mu_{n}\right)}\int_{0}^{1}J_{0}(\rho \mu_{n})d\rho$$

The average moisture content is shown in Equation (17):

(17) $$\bar{\phi }(t)=2\sum_{n=1}^{\infty }\frac{2Bi\exp(-\mu_{n}^{2}Fo)}{\left[\mu_{n}^{2}+Bi^{2}]J_{0}(\mu_{n}\right)}\sum_{m=0}^{\infty }\frac{{\left(-1\right)}^{m}}{{\left(m!\right)}^{2}(2m+1)}{\left(\frac{\mu_{n}}{4}\right)}^{2m}$$

## 5. Comparison

To verify the accuracy and applicability of the above-mentioned theoretical calculation model, the test results are compared with the theoretical calculation model. The comparison mainly involves the average moisture content of the cross-section of the log component (Equation (17)), the spatial distribution of the moisture content of the cross-section (Equation (12), with time as the known quantity), and the change in the moisture content of the cross-section over time (Equation (12), with the relative distance *ρ* of the cross-section as the known quantity).

### 5.1. Average Moisture Content

**Figure 10:**
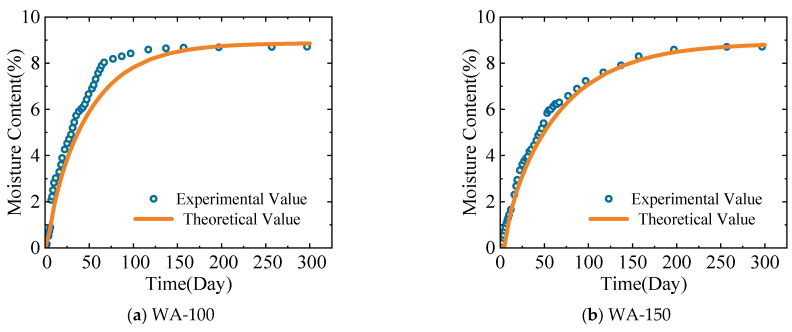

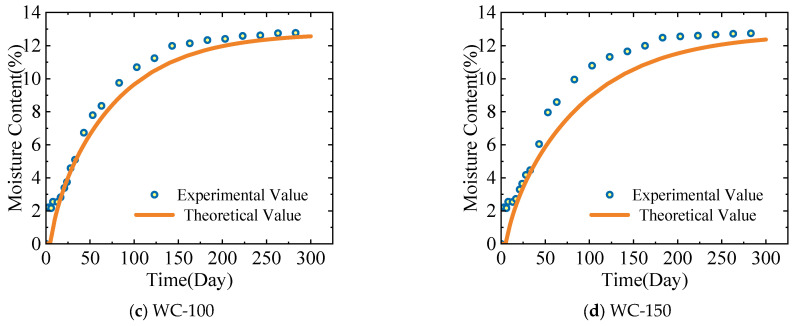
Comparison of the experimental and theoretical values of the average moisture content.

Figure 10 depicts the comparison results between the theoretical model calculation results (from Equation (12)) and the experimental results of the average moisture content of the cross-section of the specimens in the WA-100, WA-150, WC-100, and WC-150 groups.

The comparison of the experimental and theoretical values of the average moisture content in Figure 10 suggests the following. The experimental values and theoretical values show a high degree of consistency. The theoretical model proposed in this study can effectively simulate the average moisture content of wood components. For the specimens in the WA-150 group, an extremely high degree of consistency exists between the results from the experiment and the theoretical model. At the end of the experiment, the experimental values were very similar to the theoretical values.

### 5.2. Moisture Distribution in Space

**Figure 11:**
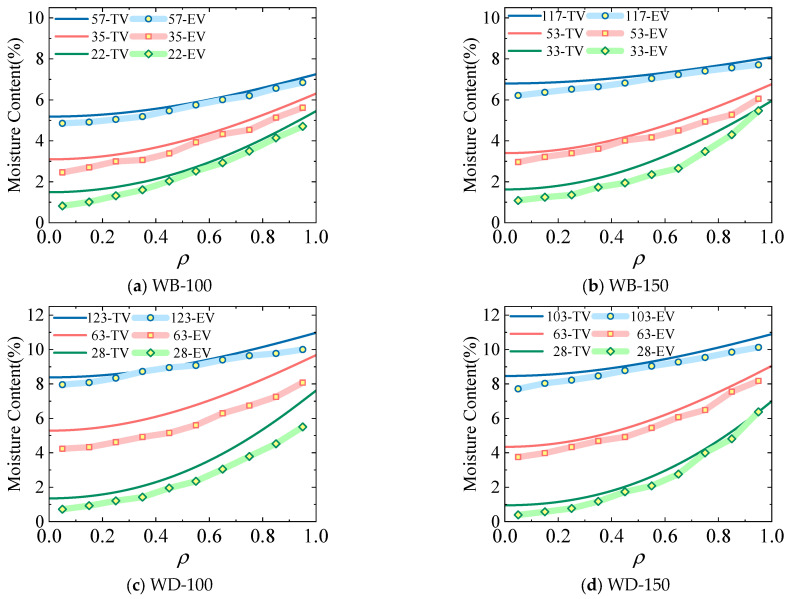
Comparison of the experimental and theoretical values of the moisture content gradient.

Figure 11 depicts the comparison results between the theoretical model (Equation (12)) calculation results and the experimental results of the spatial distribution of the average moisture content for the cross-sections of specimens in the WB-100, WB-150, WD-100, and WD-150 groups. Noticeably, when applying the theoretical calculation model in Equation (7), the time of the corresponding experimental group is taken as a known quantity, and the relative distance of the cross-section is taken as a variable to calculate the moisture content successively. In the figure, TV represents the calculated value of the theoretical model, and EV represents the average value of the experimental values. For example, 57-TV represents the calculated value of the theoretical model of the specimens in this group on the 57th day.

Comparing the experimental values and the calculated values of the theoretical model for the spatial distribution of the cross-sectional moisture content reveals a high degree of agreement between the experimental and theoretical values. The theoretical model proposed in this paper is applicable in terms of the spatial distribution of the cross-sectional moisture content of wood components. Due to the numerous cutting processes performed on the specimens during the experiment, the moisture content of the specimens is reduced, and the theoretical values are generally larger than the experimental values.

### 5.3. Moisture Distribution over Time

**Figure 12:**
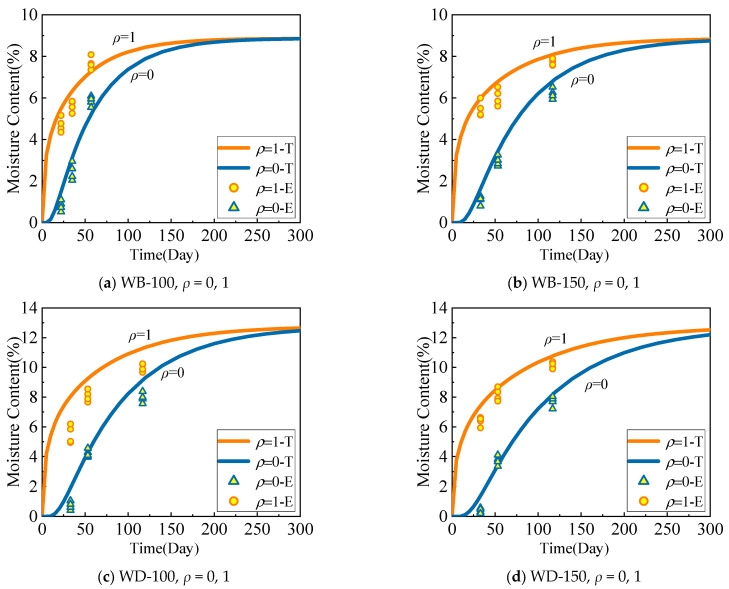

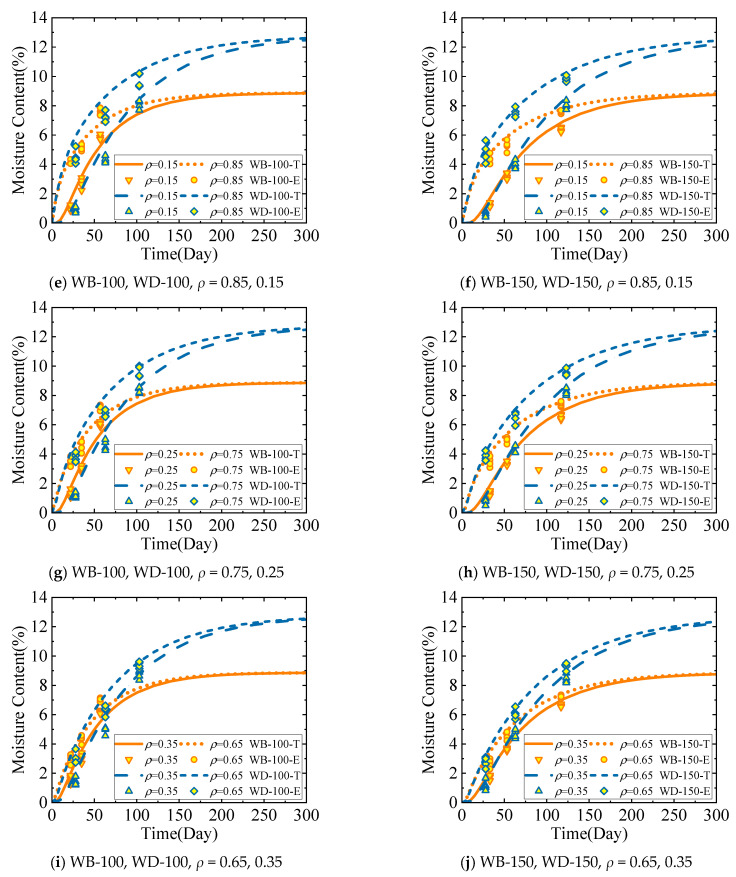

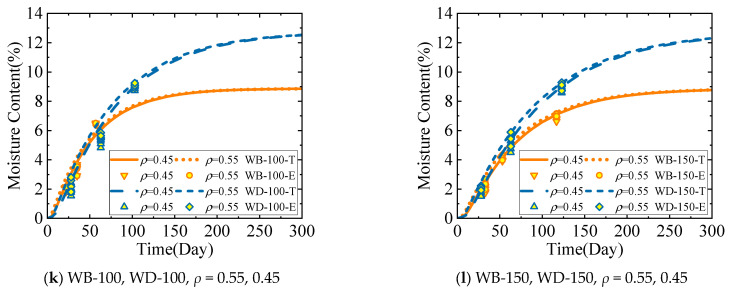
Comparison between the calculated values from the theoretical model and the test values of the moisture content of the cross-section changing with time.

Similarly, Figure 12 compares the theoretical model calculation results (Equation (12)) and the experimental results of the change in the average moisture content of the cross-section of the specimens in the WB-100, WB-150, WD-100, and WD-150 groups over time. The difference is that when applying the theoretical calculation model Equation (12), the relative cross-sectional distances are taken as known quantities, and time is taken as a variable to calculate the moisture content successively. In the figure, T represents the calculated value of the theoretical model, and E represents the average value of the experimental results.

Comparing the experimental and calculated values of the theoretical model for the change in the cross-sectional moisture content over time reveals a high degree of agreement between the two values. Therefore, the theoretical model proposed in this paper has a high degree of applicability with regard to the change in the cross-sectional moisture content of wood components over time.

## 6. Discussion

To further apply the moisture content distribution theoretical model of the cylindrical wood components, an analysis was conducted on the key parameters and important influencing factors of this model, which mainly involved the root of the characteristic equation order *μ*_n_, the component size *R*, and the cross-section relative position ratio of the component *ρ*.

### 6.1. Root of the Order μ_n_

**Figure 13:**
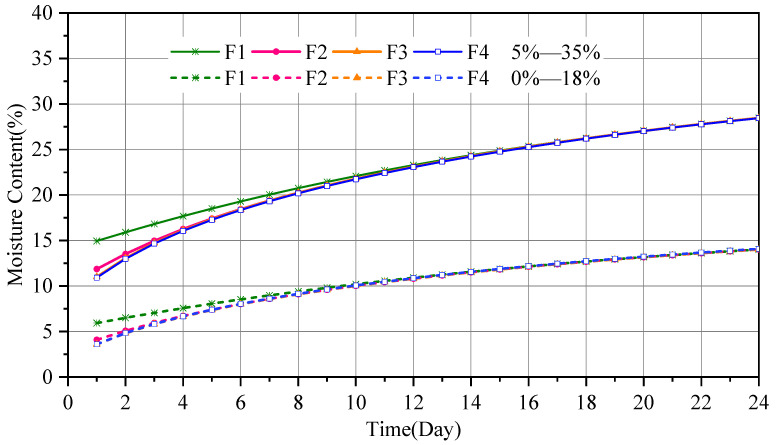
Impact of the order of *μ*_n_ on the computational precision of the average moisture content for cylindrical components.

Inspired by references [35,39], this study investigated two distinct cases of external environmental moisture content variations. The initial moisture contents were set at 0% and 5%, with corresponding final moisture contents of 18% and 35%, namely the 0–18% and 5–35% scenarios. Parameters, including the moisture diffusivity *D*, surface divergence coefficient *S*, and component size *R* were adopted from the aforementioned references. The time-dependent evolution of the average moisture content was computed by considering the first, first two, first three, and first four orders of *μ*_n_, as illustrated in Figure 13. For the 0–18% case, the first four orders of *μ*_n_ were determined as 2.3918, 5.4901, 8.6072, and 11.7149, respectively. In the 5–35% scenario, the corresponding values of the first four orders of *μ*_n_ were 2.2946, 5.2712, 8.2742, and 11.2771.

Figure 13 shows that when only the first order of *μ*_n_ is considered, relatively large calculation errors are evident in the early stage, but the errors become smaller in the later stage. Conversely, when the first three orders or the first four orders of *μ*_n_ are considered, the errors are minimal both in the early and later stages, thereby achieving a high level of accuracy. Therefore, when calculating the model for the average moisture content of cylindrical components, as well as the models depicting the spatial variation and temporal evolution of the moisture content, it suffices to adopt the first three orders of *μ*_n_.

### 6.2. Component Size R

**Figure 14:**
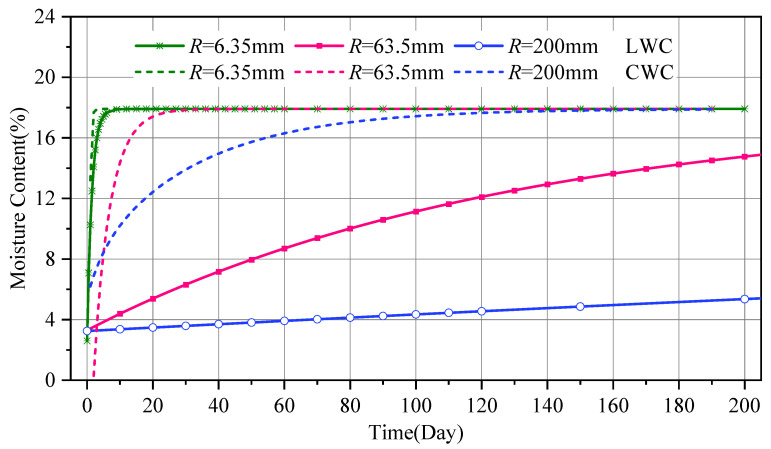
The Influence of Component Dimensions on the Average Moisture Content.

Based on the moisture content distribution prediction model, calculations were performed for laminate-type and cylindrical components, under the condition that the external moisture content increased from 0% to the equilibrium moisture content of 18%. The relevant parameters were obtained from [35,39], and the calculation results are shown in Figure 14. Noticeably, the moisture absorption rate of cylindrical components is faster than that of laminate components because the ratio of the surface area to the volume of a cylinder is larger than that of the laminate, and the former is twice that of the latter.

### 6.3. Position in the Component ρ

**Figure 15:**
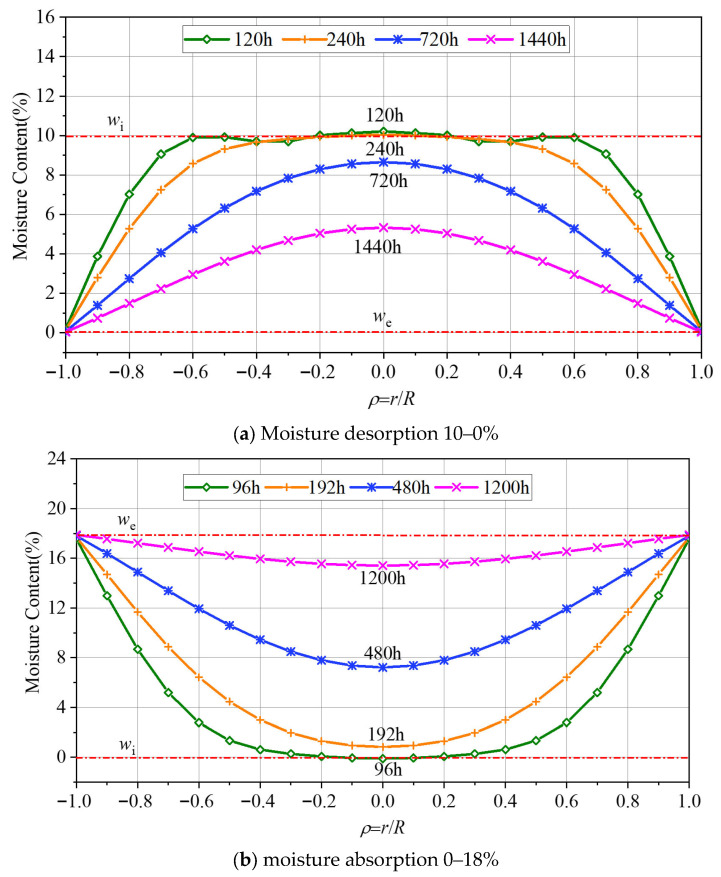
Variations in moisture content at different positions of the cross-section of cylindrical components.

Based on the prediction model of the moisture content distribution for cylindrical wood components, the variations in moisture content at different positions of the component cross-section during desorption and absorption were calculated. During moisture desorption, the external moisture content changed from 10% to 0%, and during moisture absorption, the external moisture content changed from 0% to 18%. The calculation results are presented in Figure 15. Noticeably, the position with a relative distance *ρ* = 1.0 (the surface layer) of the component can reach the equilibrium moisture content within a few hours. However, for the position with *ρ* = 0 (the innermost layer), it takes a considerably long time to reach the equilibrium moisture content. It requires approximately 70 days for it to fully attain the equilibrium moisture content.

## 7. Conclusions

In this study, the moisture content distribution model of cylindrical components was investigated through experimental tests and theoretical analyses. The study focused on the average moisture content of the components, as well as the temporal and spatial variations in the moisture content. The accuracy and applicability of the theoretical model were verified via experiments. The main conclusions drawn from this research are as follows:

(1) It is feasible to establish a moisture field model of wood components by referring to the theory of food drying and using a temperature field model. Comparison with experimental data reveals that the established time-varying moisture field model for cylindrical wood components has high accuracy. This model can provide a relatively accurate reference for predicting the change in the average moisture content of wood components, the change in moisture content over time, and its distribution law in space.
(2) During moisture absorption, the distribution of the average moisture content of wood components increased continuously with time, and the growth rate of the average moisture content gradually decreased. Both the average moisture content and the radius of the specimen affected the moisture absorption efficiency of wood components. Under similar environmental conditions, specimens with a smaller radius have a higher moisture absorption efficiency. When the radii of the specimens are the same, specimens with a lower average moisture content have a higher moisture absorption efficiency.
(3) During moisture absorption and desorption in cylindrical components, the greater the moisture content difference, the faster the equilibrium moisture content is reached. Moreover, the time for the outer part to reach the equilibrium moisture content was much shorter than that for the inner part of the component.
(4) When the external moisture content changed, the change range of the moisture content was smaller closer to the inside of the wood component, and hysteresis was more evident; the method of directly replacing the moisture content of wood components with the equilibrium moisture content of the external environment was inaccurate. At the initial stage of the experiment, the surface moisture content was high, whereas the internal moisture content was low. As the experiment progressed, the difference in the moisture content gradient between the inside and the surface of the specimen became increasingly smaller, and the moisture content of the entire cross-section of the specimen approached the equilibrium moisture content.
(5) When calculating the model for the average moisture content of cylindrical components and using the models depicting the spatial variation and temporal evolution of the moisture content, it is sufficient to take the first three orders of the roots *μ*_n_ of the characteristic equation of the Bessel function of the first type *J* in the theoretical model for long time of diffusion, which can achieve a high level of accuracy. For wood components with dimensions commonly used in engineering, when calculating their moisture content over a short period (i.e., a dozen days), a higher order of the roots *μ*_n_ of the characteristic equation of the Bessel function of the first type *J* results in greater calculation accuracy.
(6) When the diameter of cylindrical components is equal to the thickness of laminate components, the rates of moisture absorption and desorption of cylindrical components are higher than those of laminate components. Regardless of the moisture absorption or desorption process, the exterior of the components reached the equilibrium moisture content much earlier than the interior of the components.

## 8. Limitations and Future Work

Despite the contributions of this study, several limitations should be acknowledged to provide a comprehensive perspective.

(1) The experiments were only conducted on a single type of Chinese fir. This narrow scope restricts the generalizability of the results, as different wood species may exhibit distinct moisture diffusion behaviors under the same conditions.
(2) The Bessel function-based model assumes constant diffusion coefficients and constant temperature. In practical applications, however, wood diffusivity varies significantly with both moisture content and temperature, which may restrict the model accuracy in predicting moisture transfer behaviors. Additionally, the current calculation methods for the moisture diffusivity *D* and sorption coefficient *S* have limitations. The preliminary estimation equations for these parameters were originally derived for wood slabs. When applied directly to cylindrical components, these equations may introduce deviations. This further affects the prediction reliability of the model.

Future work will establish stronger links between the obtained results and practical engineering applications, such as optimizing building timber performance, improving wood preservation processes, and guiding the restoration of historical wood structures. Additionally, subsequent studies will investigate the implications of moisture-related findings for wood mechanical properties (including cracking tendency) to address this critical aspect of moisture effects in wood.

## Figures and Tables

**Table 1:** Average moisture content of the specimens.

No.	Specimen Size			Number	Environment			Test Purpose
	Radius *R* (mm)	Length *L* (mm)	Angle *θ* (°)		*T* (°C)	*RH* (%)	*W*_e_ (%)	
WA-100	100	40	45	12	30	50	8.87	MC-A
WA-150	150	40	45	12	30	50	8.87	MC-A
WC-100	100	40	45	12	30	70	12.78	MC-A
WC-150	150	40	45	12	30	70	12.78	MC-A
WB-100	100	40	45	12	30	50	8.87	MC-G
WB-150	150	40	45	12	30	50	8.87	MC-G
WD-100	100	40	45	12	30	70	12.78	MC-G
WD-150	150	40	45	12	30	70	12.78	MC-G

Note: MC-A and MC-G represent average moisture content and moisture content gradient, respectively. Twelve specimens were tested in each group and numbered starting from 1. For example, the 12 specimens in the WB-100 group are named WB-100-1, WB-100-2, WB-100-3… WB-100-12.

**Table 2:** Results of the permeability test.

No.	Direction	*m*_0_ (g)	*m*_P_ (g)	*w =* (*m*_P_ – *m*_0_)*/m*_0_ (%)
W1	Longitudinal	98.29	99.06	0.78%
W2	Tangential	185.23	187.45	1.19%
W3	Radial	134.63	135.97	0.99%

**Table 3:** Calculation results of the parameters *D* and *S.*

No.	Diameter (mm)	*w*_e_ (%)	*t* (day)	*t*_0.5_ (day)	*D* (mm^2^/day)	*S* (mm/day)
WA-100	200	8.87	86.92	24.96	78.53	1.13
WA-150	300	8.87	136.92	38.17	115.54	1.06
WC-100	200	12.78	143	40.9	47.92	0.68
WC-150	300	12.78	170	46.1	95.66	0.85

## Data Availability

The original contributions presented in this study are included in the article. Further inquiries can be directed to the corresponding author.

## Nomenclature

*w*_e_	equilibrium moisture content (%)
*w*	moisture content (%)
*T*	ambient temperature (°C)
*RH*	relative humidity (%)
*r*	radius of specimen (mm)
*L*	length of specimen (mm)
*θ*	angle of specimen (°)
*m_w_*	mass of the specimen at a certain measurement time point (g)
*m*_0_	mass of the permeability specimen at absolute dry time (g)
*m*_P_	mass of the permeability specimen at initial state (g)
*m*_1_,*m*_2_,*m*_3_..	mass of the cut specimen at initial state (g)
*m*_1_′,*m*_2_′,*m*_3_′…	mass of the cut specimen at absolute dry time (g)
EV	experimental value
MV	mean value
ρ	relative distance of the cross-section
*t*_0.5_	time required to reach half of the total adsorption capacity
*t*	time required to reach the total adsorption capacity
*D*	moisture diffusivity (mm^2^/day)
*S*	surface emission coefficient (mm/day)
*ϕ*	relative moisture content
*w*_i_	initial moisture content (%)
*r*	distance from any point to the center
*R*	half the radial length of component
*w*_s_	moisture content of the wood surface
*J*	the first-class Bessel Function
*μ*_n_	the root of the characteristic equation
*ν*	shape factor of the Bessel Function
*Bi*	Biot number for mass transfer
*Fo*	Fourier number
